# Identification and ultrastructural characterization of the *Wolbachia* symbiont in *Litomosoides chagasfilhoi*

**DOI:** 10.1186/s13071-015-0668-x

**Published:** 2015-02-04

**Authors:** Vanessa Aparecida Chagas-Moutinho, Rosane Silva, Wanderley de Souza, Maria Cristina Machado Motta

**Affiliations:** Laboratório de Biologia de Helmintos Otto Wucherer, Instituto de Biofísica Carlos Chagas Filho, Universidade Federal do Rio de Janeiro, 21941-902 Rio de Janeiro, RJ Brazil; Laboratório de Ultraestrutura Celular Hertha Meyer, Instituto de Biofísica Carlos Chagas Filho, Universidade Federal do Rio de Janeiro, 21941-590 Rio de Janeiro, RJ Brazil; Instituto Nacional de Ciência e Tecnologia em Biologia Estrutural e Bioimagens, Rio de Janeiro, Brazil; Laboratório de Metabolismo Macromolecular Firmino Torres de Castro, Instituto de Biofísica Carlos Chagas Filho, Universidade Federal do Rio de Janeiro, 21941-590 Rio de Janeiro, RJ Brazil; Diretoria de Metrologia Aplicada às Ciências da Vida, Instituto Nacional de Metrologia, Qualidade e Tecnologia- INMETRO, Duque de Caxias, RJ Brazil

**Keywords:** Filarial nematodes, *Litomosoides chagasfilhoi*, Symbiosis, Transmission electron microscopy, Ultrastructure analyses, *Wolbachia*

## Abstract

**Background:**

Filarial nematodes are arthropod-transmitted parasites of vertebrates that affect more than 150 million people around the world and remain a major public health problem throughout tropical and subtropical regions. Despite the importance of these nematodes, the current treatment strategies are not efficient in eliminating the parasite. The main strategy of control is based on chemotherapy with diethylcarbamazine, albendazole and ivermectin. In the 1970s, it was found that some filarids possess endosymbiotic bacteria that are important for the development, survival and infectivity of the nematodes. These bacteria belong to the genus *Wolbachia*, which is a widespread and abundant intracellular symbiont in worms. Knowledge about the structure of the bacteria and their relationship with their nematode hosts may allow new perspectives for the control of filarial nematodes.

**Methods:**

In this study, we used transmission electron microscopy combined with three-dimensional approaches to observe the structure of the endosymbiont of the filarial nematode *Litomosoides chagasfilhoi*, an experimental model for the study of lymphatic filariasis. In addition, the bacterium was classified based on PCR analyses.

**Results:**

The bacterium was mainly found in the hypodermis and in the female reproductive system in close association with host cell structures, such as the nucleus and endoplasmic reticulum. Our ultrastructural data also showed that the symbiont envelope is composed of two membrane units and is enclosed in a cytoplasmic vacuole, the symbiosome. Molecular data revealed that the bacterium of *L. chagasfilhoi* shares 100% identity with the *Wolbachia* endosymbiont of *Litomosoides galizai*.

**Conclusions:**

Here we described ultrastructural aspects of the relationship of the *Wolbachia* with the filarial nematode *Litomosoides chagasfilhoi* and the findings lead us to consider this relationship as a mutualistic symbiosis.

**Electronic supplementary material:**

The online version of this article (doi:10.1186/s13071-015-0668-x) contains supplementary material, which is available to authorized users.

## Background

Filarial nematodes are arthropod-transmitted parasites of vertebrates that require an intermediate host to complete their life cycle. The family Onchocercidae comprises filarial nematodes of veterinary and medical interest, as some species are causative agents of human filariasis [1,2].

Over 1 billion people in more than 90 countries are at risk of filarial nematode infections, and 150 million people are infected [3]. The major diseases caused by filarial nematodes in humans are lymphatic filariasis, filariasis and onchocerciasis [2]. Lymphatic filariasis has been identified by the World Health Organization as the second leading cause of permanent and long-term disability worldwide [4] and remains a major public health problem throughout tropical and subtropical regions [5]. Three filarial nematode species are responsible for lymphatic filariasis: *Wuchereria bancrofti*, *Brugia malayi* and *Brugia timori* [6]. Furthermore, parasitism by filarial nematodes can persist for a long time because the adult worms can live longer than a decade in a human host [7]. Thus, combatting these diseases constitutes one of the main goals of the international health community [8].

Chemotherapy remains the mainstay for the treatment of diseases caused by filarial nematodes [9]. The global program to eliminate lymphatic filariasis mainly consists of a two-drug treatment regimen capable of reducing the level of microfilaremia for long periods (1 year or more) to reduce transmission of the parasite [10]. A single yearly dose of 400 mg of albendazole (ABZ) plus 6 mg/kg of diethylcarbamazine (DEC) or 400 mg of ABZ plus 200 μg/kg of ivermectin (IVM) has been administered for a period of 4 to 6 years [11]. Despite effectiveness in the elimination of microfilariae, adult nematodes have proven to be refractory to treatment with these drugs [12]. In addition, the chemotherapy for lymphatic filariasis has not changed for the past 2–3 decades, thus producing the serious problem of drug resistance [9]. Hence, additional control strategies, such as the discovery of new drugs more effective against adult worms and new chemotherapeutic targets, are needed. It is worth mentioning that the current drug treatments are targeted to the elimination of larval microfilariae that circulate in the bloodstream or in the skin and not against the adult worms [13].

Bacterial-like structures were first observed in filarial nematodes in the 1970s by electron microscopy [7,14-17]. These prokaryotes are found in the hypodermis of male and female worms, as well as in the oocytes, embryos and larval stages [18]. The discovery of bacteria in filarial nematodes presented a new perspective for the treatment of filariasis.

Based on genomic analyses, filarial bacteria were classified as members of the *Wolbachia* genus [7,19]. Wolbachias are Alphaproteobacteria that are distributed into different clades of obligate intra-cellular rickettsiae and represent widespread and abundant endosymbiotic bacteria of arthropods and filarial nematodes [20-22]. *Wolbachia* show a diverse variety of symbiotic associations with their hosts [21]. In filarial nematodes, *Wolbachia* co-evolves in a mutualistic relationship because its presence is obligatory for normal larval growth and the development, embryogenesis and survival of adult worms [7,21,23]. Moreover, a comparison between host and bacterial genomes showed intact biosynthetic pathways for heme, nucleotides, riboflavin and FAD in the prokaryotes, indicating that the symbiont furnishes essential nutrients to the nematode host, thus influencing its physiology [3,7,21,24].

As the presence of *Wolbachia* is essential to the development and survival of its filarial hosts, the understanding of the association between the symbiont and nematode represents a new perspective for identifying possible targets for efficient filariasis treatment. Thus, in this work, our purpose is to classify the endosymbiont of the filarial nematode *Litomosoides chagasfilhoi* [25] and to characterize the relationship by ultrastructural approaches. Because of the parasite-host specificity observed in several species of filarial nematodes, research on human filariasis faces the ethical concerns in obtaining the parasites. In this sense, *Litomosoides chagasfilhoi* represents an experimental model for the investigation of lymphatic filariasis given the successfuladaptation of its life cycle in laboratory conditions [10].

## Methods

### Parasite material

The life cycle of the filarial nematode *Litomosoides chagasfilhoi* was maintained in the Laboratório de Biologia de Helmintos Otto Wucherer – Universidade Federal do Rio de Janeiro (UFRJ) using Mongolian gerbils (*Meriones unguiculatus*) as a definitive host and mites (*Ornithonyssus bacoti*) as an intermediate host. The experimental life cycle was according to [26]. All the procedures where submitted to the ethics committee of animal use of UFRJ (CEUA- Protocol 10-03/1993).

Adult worms were collected from the abdominal cavity of experimentally infected Mongolian gerbils, washed in 0.9% NaCl solution and processed according to each technique utilized.

### Molecular analyses

For the molecular analyses, adult nematodes were collected from abdominal cavities and frozen at −20°C. DNA was extracted using the phenol/chloroform method.

PCR screening were performed in a 100 μl final volume under the following conditions: 1× PCR buffer, 3 mM MgCl_2_, 250 μM dNTP mix, 100 pM of each primer and 0.2 U of Taq DNA polymerase. The thermal profile used was 30 cycles of 94°C for 30 sec, 55°C for 30 sec and 72°C for 30 sec. Primers amplifying the 16S rDNA of *Wolbachia* were used (Table 1).

**Table 1:** **Endosymbiont of** ***Litomosoides chagasfilhoi*** **(this work); DQ408758, Gammaproteobacterium endosymbiont of** ***Astomonema sp*** **. clone LSI-A2; DQ408757, Gammaproteobacterium endosymbiont of** ***Astomonema sp*** **. clone LSI-A1; DQ314214, Candidatus Paenicardinium endonii; AY278355,** ***Wolbachia*** **endosymbiont of** ***Mansonella perstans*** **isolate M02-052N; AJ548800,** ***Wolbachia pipientis*** **, specific host** ***Litomosoides galizai*** **; AJ548799,** ***Wolbachia pipientis*** **, specific host** ***Litomosoides brasiliensis*** **; AJ279034;** ***Wolbachia*** **endosymbiont of** ***Mansonella ozzardi*** **; AJ276500,** ***Wolbachia*** **endosymbiont of** ***Dirofilaria repens*** **; AJ276499,** ***Wolbachia*** **endosymbiont of** ***Onchocerca gibsoni*** **; AJ012646,** ***Wolbachia*** **endosymbiont of** ***Brugia pahangi*** **; AF069068; Endosymbiont of** ***Litomosoides sigmodontis***

	**AJ279034**	**AY278355**	**AJ276500**	**AJ276499**	**DQ408758**	**DQ408757**	**DQ314214**	***L.chagas filhoi***	**AF69068**	**AJ548800**	**AJ548799**	**AJ012646**	**AJ279034**
**AJ279034**													
**AY278355**	99.86												
**AJ276500**	96.60	96.74											
**AJ276499**	96.60	96.45	98.01										
**DQ408758**	78.62	78.62	78.48	78.76									
**DQ408757**	78.62	78.62	79.04	79.32	97.16								
**DQ314214**	74.26	74.26	73.69	73.69	73.60	74.16							
***L. chagasfilhoi***	97.87	97.73	96.74	96.74	78.48	78.76	74.26						
**AF69068**	97.45	97.30	96.31	96.31	78.06	78.34	74.12	**99.57**					
**AJ548800**	97.87	97.73	96.74	96.74	78.48	78.76	74.26	100.00	99.57				
**AJ548799**	97.45	97.30	96.03	96.60	77.64	77.92	73.55	98.72	98.30	98.72			
**AJ012646**	97.30	97.16	96.88	96.88	78.48	78.76	74.54	98.58	98.16	98.58	97.87		
**AJ279034**	100.0	99.86	96.60	96.60	78.62	78.62	74.26	97.87	97.45	97.87	97.45	97.30	

The PCR products were purified with the Amicon® Ultra-0.5 30 K kit and sequenced using the Big Dye kit and the ABI 3500 Genetic Analyzer (Life Technologies). The primers used for PCR screening were also used for sequencing the samples, as indicated in Table 1. The reads were assembled using Geneious Software (v.6). The consensus 16S rRNA sequence was searched against GenBank using Blastn and aligned to other nematode endosymbionts. A phylogenetic tree was generated using CLC Genomic Workbench Software (v.7). The parameters were the Neighbor Joining method and HKY nucleotide substitution model with 100 bootstrap replications.

### Ultrastructural analyses

#### Transmission electron microscopy (TEM)

For TEM, adult nematodes were fixed in a solution containing 2.5% glutaraldehyde and 4% freshly prepared formaldehyde diluted in 0.1 M sodium cacodylate buffer, pH 7.2. After fixation, the nematodes were washed in 0.1 M sodium cacodylate buffer, pH 7.2, and post-fixed in a solution containing 1% osmium tetroxide and 0.8% potassium ferrocyanide, pH 7.2, for 1 hour. The samples were then dehydrated in a graded series of acetone and embedded in Epoxy resin. Thin sections were collected on copper grids, counterstained with uranyl acetate and lead citrate and observed with a Jeol 1200 transmission electron microscope.

#### Ultrastructural cytochemistry – osmium tetroxide-potassium iodide

Adult nematodes were fixed as described above and then washed twice in a solution containing 1% potassium iodide diluted in 0.1 M sodium cacodylate buffer, pH 7.2. The samples were post-fixed overnight in 1% osmium tetroxide and 1% potassium iodide diluted in 0.1 M sodium cacodylate buffer. After this procedure, the samples were washed again in the solution containing 1% potassium iodide, dehydrated in a graded series of acetone and embedded in Epoxy resin. Thin sections were collected on copper grids, counterstained with 1% lead citrate and observed with a Jeol 1200 transmission electron microscope.

#### Electron tomography (ET)

For ET, semi-thin sections (200 nm) or ribbons of serial sections were collected on Formvar-coated slot copper grids. The samples were post-stained with uranyl acetate and lead citrate, incubated with 10-nm colloidal gold particles on both sides for 5 min and washed in distilled water. The sections were observed using a 200 kV transmission electron microscope (Tecnai G2, FEI Company, Eindhoven) equipped with a 4 k × 4 k CCD camera (Eagle, FEI Company, Eindhoven) that was used to record the tomograms. Tilt series from – 60° to + 60° with an angular increment of 1° were used to acquire all tomograms.

##### Three-dimensional reconstruction

A 200 kV transmission electron microscope (Tecnai G2, FEI Company, Eindhoven) equipped with a 4 k × 4 k CCD camera (Eagle, FEI Company, Eindhoven) was used to record tomograms. Models were constructed on a computer running MIDAS and IMOD software (Boulder Laboratory, University of Colorado, Boulder, Colorado, USA). Image stacks were aligned using MIDAS. IMOD was used to stack the aligned images, and the structures of interest were traced to provide a 3-D representation. Using the IMODmesh feature of IMOD, the contours of each object were joined to form a 3D model. Movies of these models rotating in space were generated using Quick Time software.

## Results

**Figure 1:**
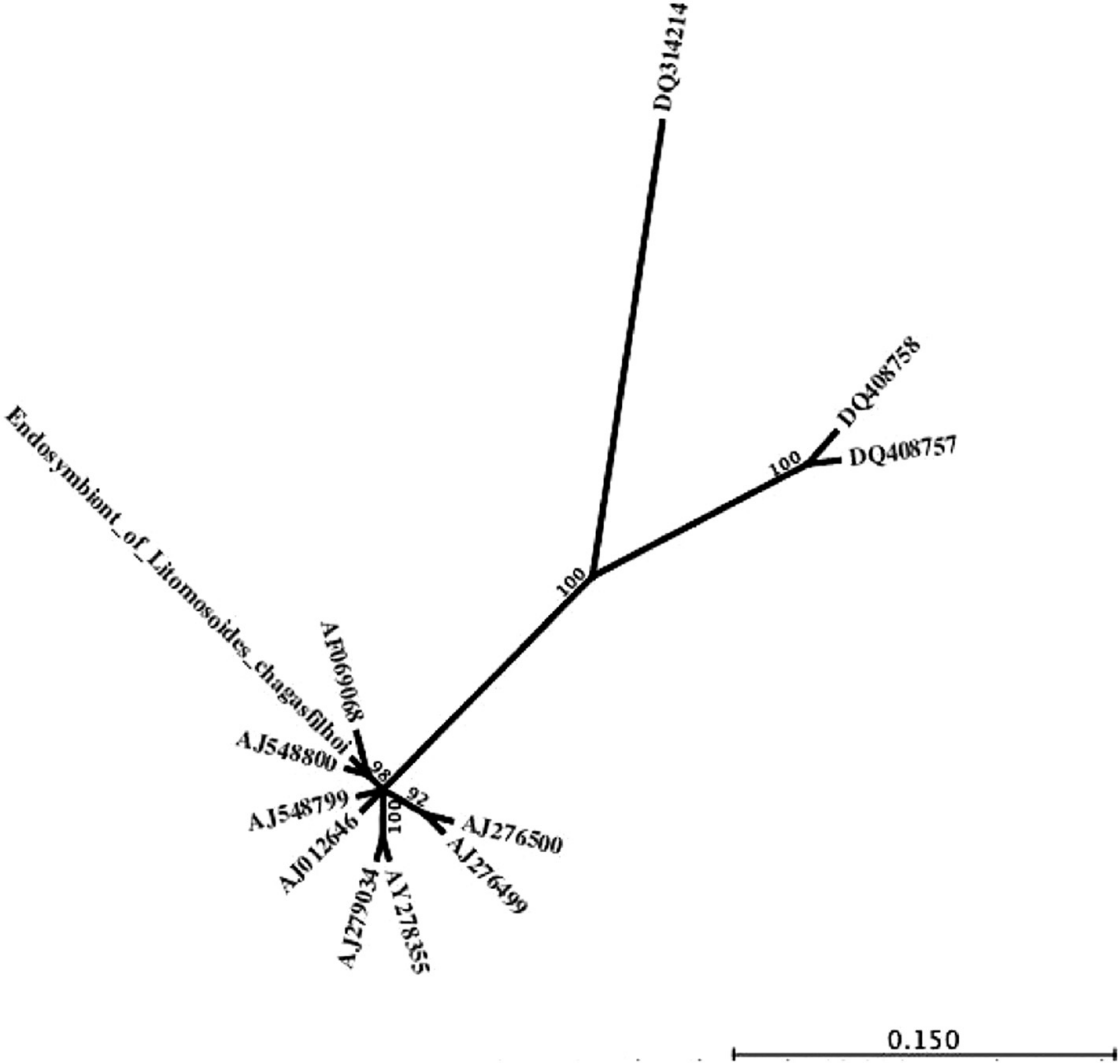
**Radial phylogenetic tree of 12 nematode endosymbionts species based on the analysis of 705 nucleotides of 16S rDNA.** Bootstrap values >90 are indicated. Endosymbiont of *Litomosoides chagasfilhoi* (this work); **DQ408758**, Gammaproteobacterium endosymbiont of *Astomonema sp*. clone LSI-A2; **DQ408757**, Gammaproteobacterium endosymbiont of *Astomonema sp*. clone LSI-A1; **DQ314214**, Candidatus Paenicardinium endonii; **AY278355**, *Wolbachia* endosymbiont of *Mansonella perstans* isolate M02-052 N; **AJ548800**, *Wolbachia pipientis*, specific host *Litomosoides galizai*; **AJ548799**, *Wolbachia pipientis*, specific host *Litomosoides brasiliensis*; **AJ279034**, *Wolbachia* endosymbiont of *Mansonella ozzardi*; **AJ276500**, *Wolbachia* endosymbiont of *Dirofilaria repens*; **AJ276499**, *Wolbachia* endosymbiont of *Onchocerca gibsoni*; **AJ012646**, *Wolbachia* endosymbiont of *Brugia pahangi*; **AF069068**, Endosymbiont of *Litomosoides sigmodontis.*

Using the total DNA extracted from adult females of *L. chagasfilhoi*, it was possible to amplify one fragment of 16S rDNA from the endosymbiotic bacterium. Based on this fragment, a sequence of 896 bp was obtained, which showed an identity of 100% with the *Wolbachia* endosymbiont of *Litomosoides galizai* (accession number: AJ548800) (Figure 1). Table 1 shows a pairwise comparison of the symbiont with other species of filarial nematodes.

**Figure 2:**
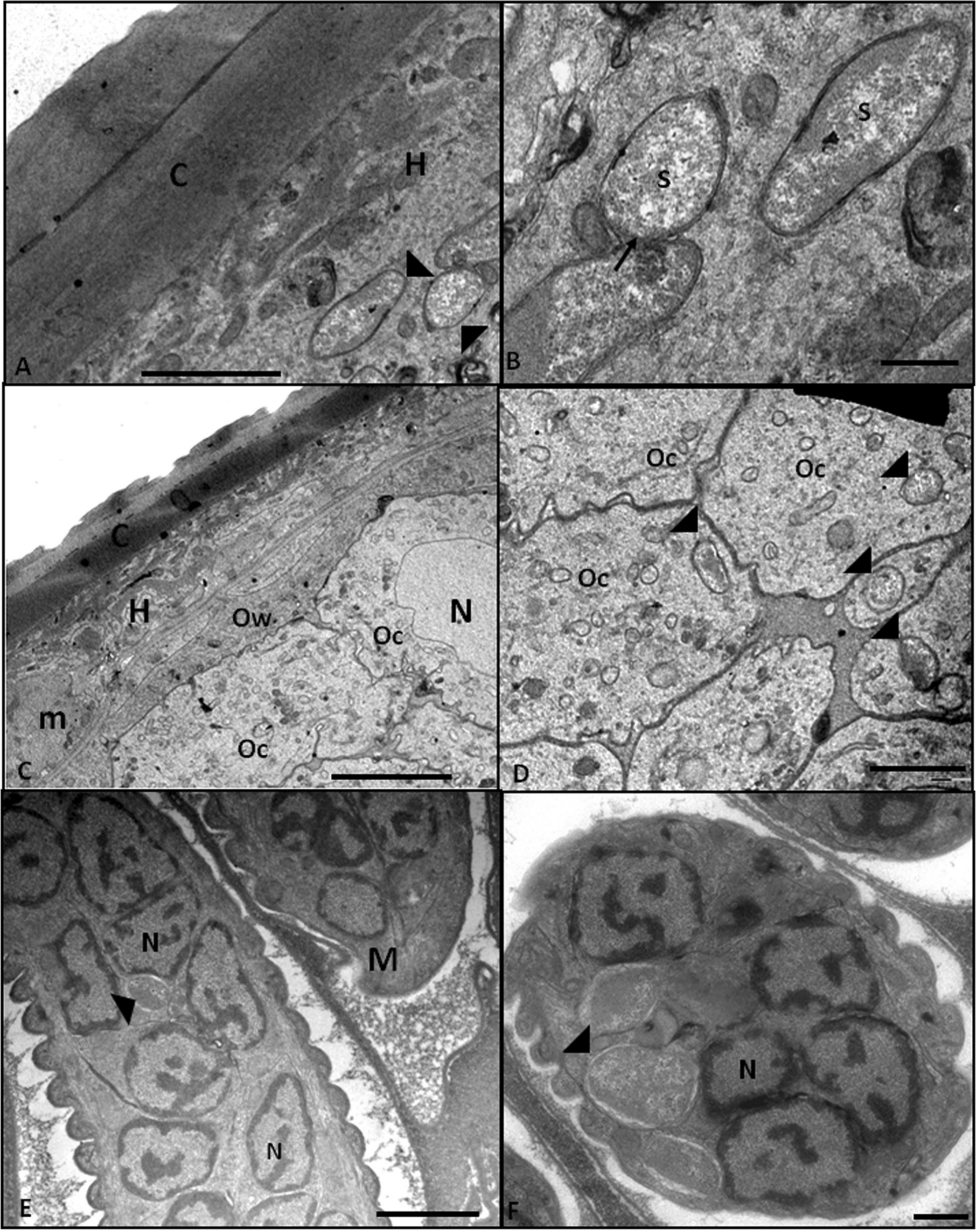
**Transmission electron microscopy revealing that the symbiotic bacterium is present in different tissues of** ***Litomosoides chagasfilhoi.*** **A **– General view of the body wall showing the cuticle (C) and a bacterium (black arrowhead) present on the hypoderm (H) (Bar 2 μm). **B **– At high magnification, it is possible to observe a dividing bacterium on the hypoderm. It is worth noting that a classical septum is absent in the central constriction region (arrow) (Bar 5 μm). **C** and **D **– Transversal section showing the body wall composed of the cuticle (C), hypoderm (H), muscle layer (m) and proximal region of the ovary showing the ovary wall (Ow) and oocytes (Oc). At higher magnification, the endosymbiotic bacteria (arrowheads) are observed in the cytoplasmic oocytes and nucleus (N) (Bar = 50 μm in C and 20 mm in D). **E **– Longitudinal view of microfilariae (M) showing the nucleus (N) of sub-cuticular cells and endosymbiotic bacteria (arrowheads). Bar = 2 μm. **F **– At higher magnification, it is possible to note that in the microfilariae (M), the endosymbiont is inside a vacuole (arrowhead) close to the host cell nucleus (N) (Bar 0.5 μm.)

**Figure 3:**
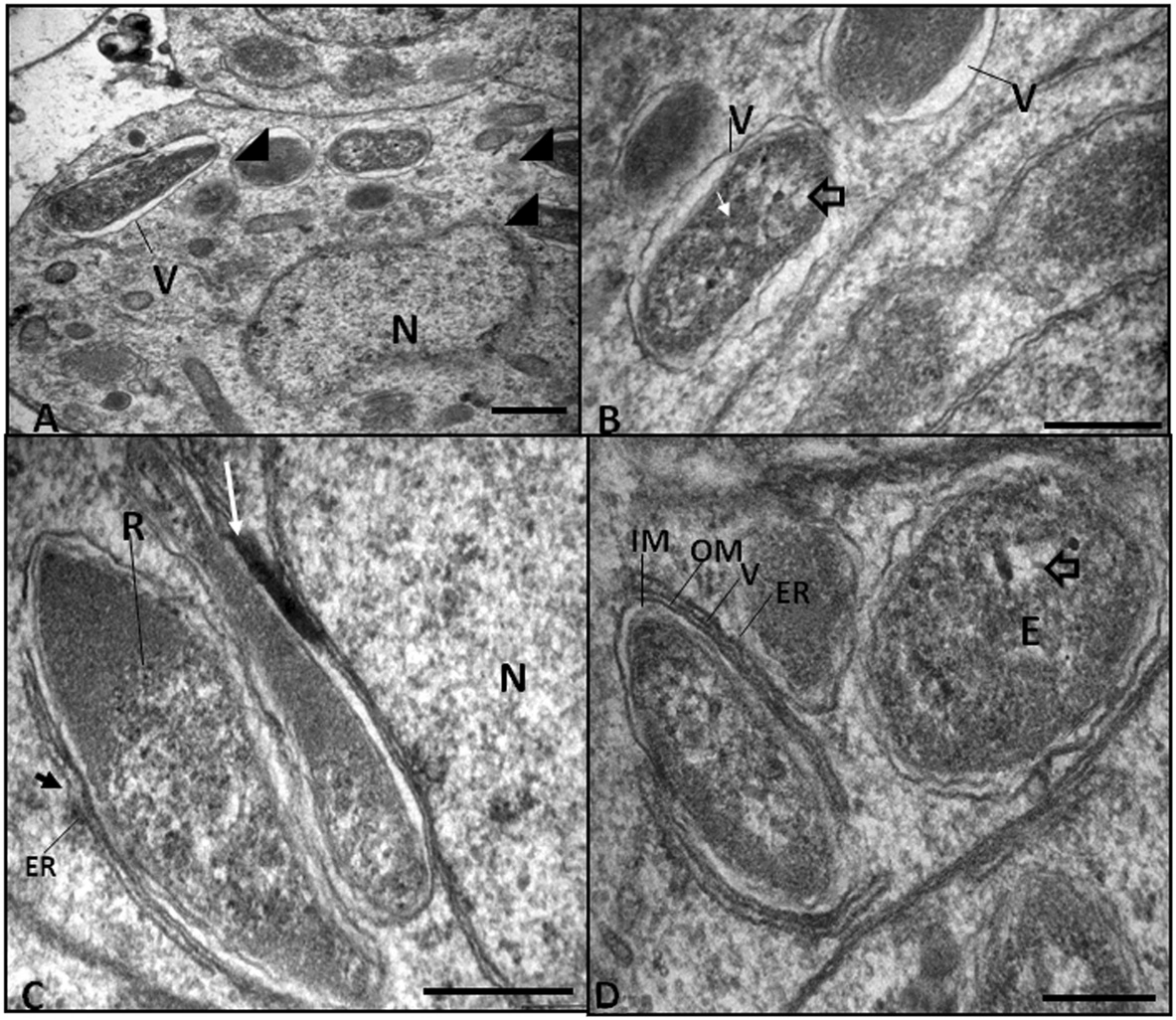
**Transmission electron microscopy showing the** ***Litomosoides chagasfilhoi*** **symbiont ultrastructure. A - D**: symbionts observed in early-embryo stages. (A) Endosymbiotic bacteria (arrowheads) were observed inside a vacuole (V) and close to the nucleus (N). (B) The symbiont matrix is composed of an electron-dense region rich in ribosomes (small white arrow), and an electron-lucid area that contains the DNA fibers (f – open arrowhead) (Bar 0.5 μm). **C **– At high magnification, it is possible to observe the symbiont in close proximity to the host cell nucleus (N). An electron-dense region is indicative that the symbiont vacuole touches the nuclear envelope (white arrow). It is also interesting to note the association (small black arrow) between the symbiont and the endoplasmic reticulum (RE) containing ribosomes (R). (Bar 0.5 μm). **D **– The endosymbiont is enclosed by two membrane units: an outer membrane (OM) that faces the vacuole (V) and an inner membrane (IM) that faces the matrix. Note that the symbiont electron-lucid area in the matrix contains DNA fibers (open arrowhead) and that the bacteria are associated with the endoplasmic reticulum (R) with associated ribosome (Bar 0.5 μm).

Ultrastructural analyses revealed numerous bacteria in some regions of the hypoderm (Figure 2A-B). In some sections, it was possible to observe a dividing symbiont that did not present a typical septum (Figure 2B), indicating that, similar to other intracellular bacteria, this endosymbiont presents a reduced cell wall. Bacteria were also observed in oocytes (Figure 2C-D), in early-stage embryos (Figure 3A-D) and in completed developed intrauterine microfilariae (Figure 2E-F). The TEM analyses demonstrated that the bacterium was located close to the host cell nucleus in embryo cells and in microfilariae (Figure 2E-F and Figure 3A-C).

During the ultrastructural analyses of different filarial tissues, endosymbiotic bacteria were found in an intracellular vacuole, the membrane of which was juxtaposed to the symbiont envelope (Figures 3A-D). Endosymbionts were typically observed close to the host cell nucleus (Figure 3A). The symbiont matrix presented an electron-dense region containing ribosomes and also an electron-lucid area containing DNA fibers (Figures 3B). In some thin sections, it was possible to observe an electron-dense region between the symbiont vacuole and the nuclear envelope, indicating an association between both structures (Figures 3C). The bacterium was also observed in close proximity with profiles of rough endoplasmic reticulum (Figures 3C-D). A symbiont located in a vacuole was enclosed by two membrane units: an outer membrane that faces the vacuole and an inner membrane in contact with the bacterial matrix (Figure 3D).

**Figure 4:**
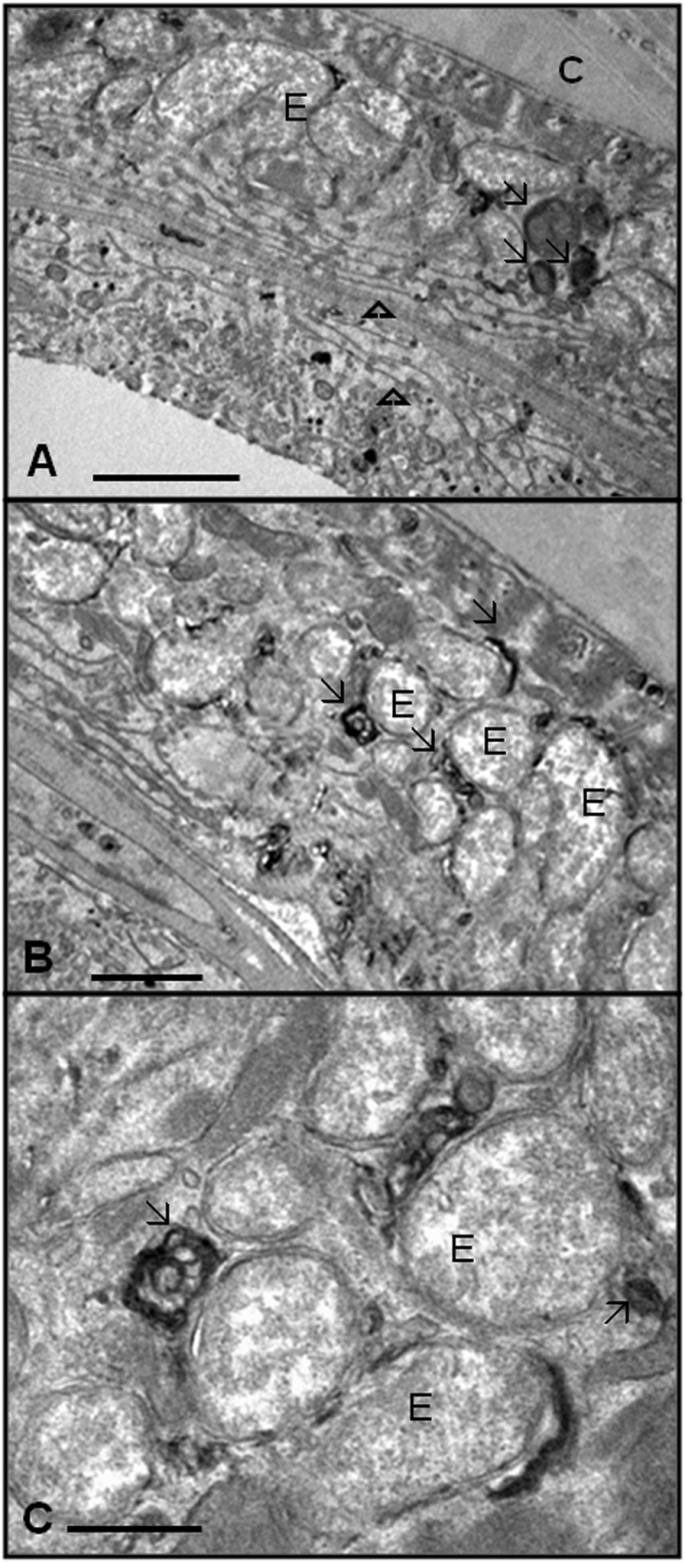
**Ultrastructural cytochemistry of** ***Litomosoides chagasfilhoi*** **by OsKI staining. A **– General view of the body wall showing the cuticle (C) and hypoderm (H) that present the endosymbionts (E). There is a positive reaction specific to regions near the bacteria (thin arrow) (Bar 20 μm). **B **– Note the electron-dense and reticular structures close to the endosymbiont (thin arrow) (Bar 20 μm). **C **– Detail showing the intimate association between the bacterial membrane and the electron-dense structures that resemble the membranes of the endoplasmic reticulum (thin arrow) (Bar 10 μm).

To confirm that the endosymbiotic bacterium was associated with the endoplasmic reticulum, we performed ultrastructural cytochemistry using the osmium-potassium iodide technique [27]. This reaction preferably stains glycoproteins that are being processed along the rough endoplasmic reticulum and the Golgi complex cisternae. The results showed an electron-dense staining, which was compatible with a positive reaction in reticular structures located near the bacterium-containing vacuole, thus confirming the association between the endosymbiont and the endoplasmic reticulum (Figure 4A-C).

**Figure 5:**
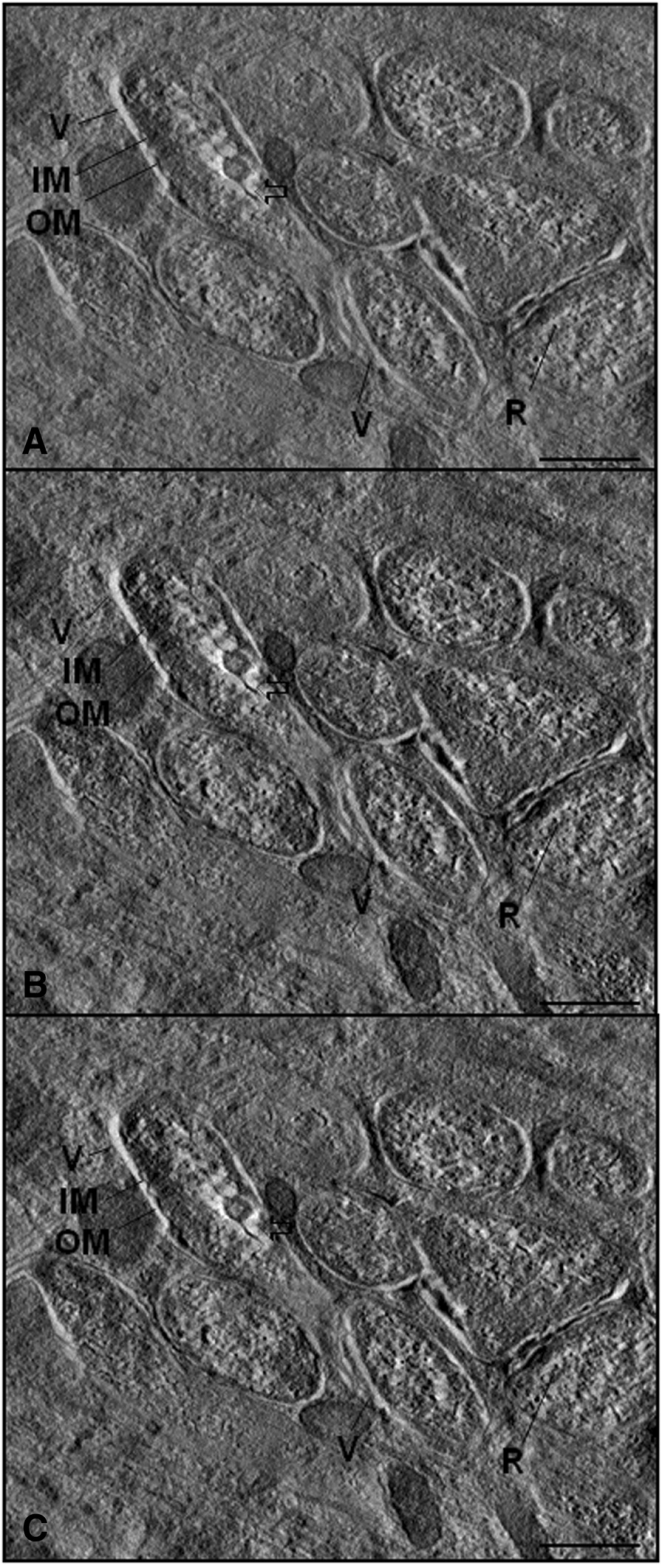
**Electron tomography of endosymbiotic bacteria of** ***Litomosoides chagasfilhoi.*** **A-C **– Tomographic series of the bacteria present in the hypodermis, showing the vacuole membrane (V), outer membrane (OM) and regions that present DNA fibers (large arrow) and ribosomes (R) (Bar 5 μm).

**Figure 6:**
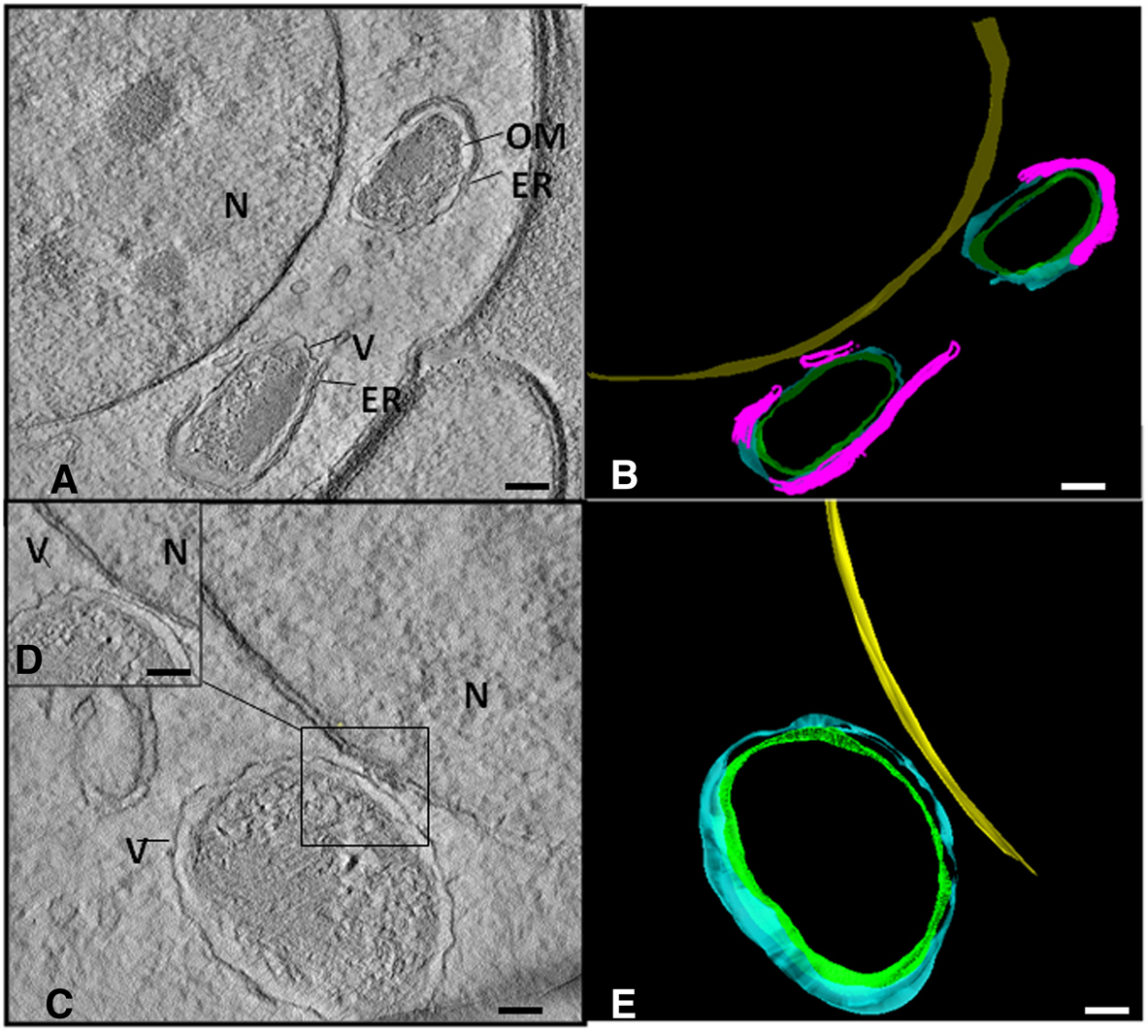
**Electron tomography and 3-D model of endosymbiotic bacteria of** ***Litomosoides chagasfilhoi.*** **A**: Tomographic series showing a bacterium in an embryo cell. The bacterium is located in a vacuole (V) near the nucleus (N), and it was possible to observe the outer membrane (OM) and some association with the endoplasmic reticulum (ER) (Bar 5 μm). **B**: 3-D model obtained from a tomographic series (yellow: nucleus, green: symbiont outer membrane, light blue: vacuole membrane and pink: endoplasmic reticulum). **C**: Tomographic series showing a bacterium and the vacuole membrane (V) in close association with the nucleus (N) of the nematode host (Bar 5 μm). **D**: Detail of the vacuole (V) and nucleus (N) proximity (Bar 5 μm). **E**: 3-D model obtained from a tomographic series (yellow: nucleus, green: symbiont outer membrane and light blue: vacuole membrane).

Analyses by TEM suggested a single bacterium per vacuole. Tomographic analyses followed by 3-D reconstruction of the hypodermis confirmed this hypothesis (Figure 5A-C and Additional file 1: Movie S1). In addition, electron tomography showed with high resolution a symbiont lacking a typical envelope and the matrix composed of DNA fibers and ribosomes (Figure 5A-C). The tomographic reconstruction of embryo cells showed that the bacterium envelope touches the nuclear membrane (Figure 6A and Additional file 2: Movie S2 and Additional file 3: Movie S3) as well as the endoplasmic reticulum of the embryo cells (Figure 6B and Additional file 4: Movie S4).

## Discussion

A bacterium-like microorganism was previously reported in the reproductive system of the filarial nematode *L. chagasfilhoi* based on ultrastructural analyses [4]. The bacterium was observed in adult females in the hypodermal cord, oocytes, zygotes and embryos. These findings corroborate those described in the literature for the location of bacteria in most filarial nematodes and are in accordance to the observations in the present study.

The presence of symbiotic bacteria in the reproductive system of filarial nematodes is related to vertical transmission to the next generation through infected oocytes [16,18,27]. Bacteria were also reported on the intestine wall of the filarial nematodes *Mansonella* (*Cutifilaria*) *perforate* and *Madathamugadia hiepei* [28,29]. Furthermore, the entomopathogenic nematode *Steinernema* Travassos, 1927 (Rhabditida: Steinernematidae) harbors the bacterial symbiont *Xenorhabdus* sp. in a modified structure known as the bacterial receptacle present in the anterior portion of the nematode intestine [30]. However, in this work, symbiotic bacteria were not identified in the intestine of *L. chagasfilhoi*.

In the present study, we performed the first ultrastructural analysis of the endosymbiont of *L. chagasfilhoi*. After observing different tissues of the host nematode, it was possible to conclude that the bacterium inhabits a host-derived vacuole and is enclosed by two membrane units, as observed for other filarial endosymbionts and also in *Wolbachia*-containing insects [16,31-34]. Some trypanosomatid protozoan species also harbor an endosymbiont, but in these cases, the Gram-negative bacterium is free in the host cytosol [35]. The fact that the symbiont of filarial nematodes lives in a vacuole can represent a strategy of the host to control the number of bacteria. As previously discussed, autophagy is a mechanism used to control the *Wolbachia* population in filarial nematodes [21].

The close association between endosymbiotic bacteria and host cell organelles has been described [36]. In the present study, we found *Wolbachia* in intimate contact with the nematode endoplasmic reticulum and also with the nucleus. Voronin et al. (2004) [33] reported that the outer membrane of the *Wolbachia* endosymbiont of *Drosophila melanogaster* embryos has continuity with the host endoplasmic reticulum. Studies on the genome of the filarial nematode *Brugia malayi* and its endosymbiont revealed that the nematode shows a metabolic dependence on this bacterium because *Wolbachia* is responsible for the synthesis of essential metabolites including heme, riboflavin, flavin adenine dinucleotide and nucleotides [3,7,37]. In the present work, the association between the symbiont and the host endoplasmic reticulum suggests that metabolic exchanges may occur between the partners, which most likely contributes to maintain this mutualistic relationship.

The symbiont of *L. chagasfilhoi* was generally observed close to the nucleus in the nematode cells, with some ultrastructural evidence that the symbiont vacuolar membrane touches the nuclear envelope. In the trypanosomatid *Angomonas deanei*, an intimate association is observed between the symbiont and the protozoan nucleus and is related to the coordinated division of the bacterium with other host cell structures. Such an association may be related to the fact that each daughter cell inherits a single symbiont at the end of the cell cycle [35]. This strategy of synchronized division is also observed in organelles of symbiotic origin, as reported for the mitochondrion and the plastid of the unicellular alga *Cyanidioschyzon merolae* [38]. Another example is the association of the apicoplast, a characteristic organelle of apicomplexan protozoa that descends from red algae, the segregation of which depends on an association with the centrosome [39]. In the present work, the close proximity of the endosymbiont with the nucleus of the nematode cells indicates that the host displays some mechanism to control the number of bacteria, as observed in many cases of obligatory symbiosis.

Despite the similar ultrastructural characteristics and distribution pattern of the *L. chagasfilhoi* symbiont in relation to other bacteria of filarial nematodes, it was necessary to perform molecular analyses to classify this symbiotic bacterium. Sequencing of the symbiont 16S rDNA revealed that this bacterium belongs to the *Wolbachia* genus. *Wolbachia* are likely the most common endosymbiont of invertebrates [22].

Endosymbiosis is a specific type of symbiosis in which one partner lives within its host, representing the most intimate contact between interacting organisms [40]. The presence of *Wolbachia* is well described in arthropods and filarial nematodes [41,42]. In arthropod hosts, the endobacteria are considered reproductive parasites, whereas in nematode hosts, *Wolbachia* display features of an obligate association characterized as mutualistic [42-44]. In filarial nematodes, the *Wolbachia* endosymbiont is transmitted vertically from mother to offspring [42,44]. However, it is not clear how this transmission occurs because it involves numerous and complex events. Sacchi (2004) [34] describe the transmission of the *Wolbachia* of the cockroach *Mastotermes darwiniensis* from the ovaries to embryo cells. In this case, the bacteria were carried out from the ovary cells via exocytosis and were then phagocytized by the embryo cells. In *L. chagasfilhoi*, numerous bacteria were found in the raquis, and we can speculate that the symbionts are transmitted from this region to the nematode germ cells.

Several studies suggests that *Wolbachia* are essential to development and survival of filariids [1,19,23,28]. In this sense, the use of tetracycline leads to depletion of the symbiotic bacterium which results in female infertility [1,2,7,13]. Our ultrastructural data revealed the intimate association between *Wolbachia* and the host nematode structures, thus reinforcing the idea that this is an obligatory relationship and that the symbiont represents an excellent chemotherapic target in filariasis treatment.

## Conclusions

Here we described ultrastructural aspects of the relationship of the *Wolbachia* with the filarial *Litomosoides chagasfilhoi* and the close association between the symbiotic bacterium and the nematode host lead us to consider this relationship as a mutualistic symbiosis.

## Additional files

Additional file 1:
***Wolbachia*** **on hypodermis.** Tomogram of a group of bacteria on the hypoderm o *Litomosoides chagasfilhoi* showing one symbiont per vacuole. Additional file 2:
***Wolbachia*** **association with host nucleus.** Tomogram showing a bacterium of an embryo cell. The symbiont is located in a vacuole and is close to the nematode host nucleus. Additional file 3:
**3D model of association of** ***Wolbachia*** **and host nucleus.** 3D reconstruction of a bacterium of an embryo cell. The symbiont is located in a vacuole (light blue) and is close to the nematode host nucleus (yellow). Additional file 4:
***Wolbachia*** **association with host nucleus and endoplasmic reticulum.** 3D reconstruction of a bacterium of an embryo cell showing the interaction with endoplasmic reticulum of the nematode host (yellow: nucleus, green: symbiont outer membrane, light blue: vacuole membrane and pink: endoplasmic reticulum).
